# Multifocal Langerhans cell histiocytosis involving the thyroid and several organs: a diagnostic dilemma

**DOI:** 10.1210/jcemcr/luag201

**Published:** 2026-07-22

**Authors:** Monwarul Abedin Khan, Mushtaq Mutashid Muhib

**Affiliations:** Department of ENT, East West Medical College and Hospital, Dhaka 1230, Bangladesh; Intern Doctor, East West Medical College and Hospital, Dhaka 1230, Bangladesh

**Keywords:** Langerhans cell histiocytosis, thyroid malignancy, head and neck malignancy, salivary gland tumor, multifocal LCH

## Abstract

Langerhans cell histiocytosis (LCH) is a rare clonal proliferative disorder of antigen-presenting cells (dendritic cells) with variable clinical presentation. Involvement of the thyroid gland, parotid gland, lungs, and salivary glands is exceedingly rare and often mimics malignancy, such as lymphoma or metastatic disease. It can closely mimic other thyroid malignancies. Immunohistochemistry remains crucial for diagnosis. Early recognition prevents unnecessary radical surgery and allows tailored multidisciplinary management. We report a 42-year-old woman with long-standing hypothyroidism presenting with bilateral thyroid swelling and submandibular and parotid enlargement. Imaging suggested chronic thyroiditis. High-resolution computed tomography of the chest revealed bronchiectasis. Total thyroidectomy was performed for diagnostic clarification. Immunohistochemistry revealed a diffuse population of CD1a- and CD68-positive cells, confirming LCH. This represents a rare case of multifocal head and neck LCH involving both thyroid lobes, the submandibular and parotid glands, and the lungs.

## Introduction

Langerhans cell histiocytosis (LCH) is a rare disorder characterized by the clonal proliferation of Langerhans-type dendritic cells. Thyroid involvement in LCH is exceptionally uncommon, and concurrent pulmonary involvement is even rarer, with only approximately 60 to 70 cases reported in the literature to date [1-3]. Recent studies have identified recurrent activating mutations in the mitogen-activated protein kinase (MAPK) pathway, including *BRAF* V600E, *MAP2K1* (MEK1), and *BRAF* N486_P490 variants, which play a key role in disease pathogenesis [1, 4]. The disease may involve single or multiple organ systems, most commonly affecting the bones, skin, and lungs, while thyroid involvement remains rare (approximately 6.6%) [5, 6]. Clinically, LCH often presents as bone or cutaneous lesions; in pediatric populations, it may manifest with recurrent otitis media or mastoid involvement. When the thyroid gland is affected, the condition can closely mimic other thyroid disorders, particularly primary thyroid lymphoma or autoimmune thyroiditis. In addition, pulmonary involvement is frequently asymptomatic, further contributing to diagnostic difficulty [4, 6]. Langerhans cell histiocytosis may also be associated with endocrine dysfunction, most notably arginine vasopressin deficiency (AVP-D) due to pituitary involvement.

Herein, we report an unusual case of multifocal head and neck LCH involving the thyroid, submandibular, and parotid glands, along with pulmonary involvement, initially suspected to represent autoimmune thyroiditis or non-Hodgkin lymphoma.

## Case presentation

A 42-year-old woman presented at the ear, nose, and throat department of East West Medical College, Dhaka, with neck swelling and swelling of the left lateral aspect of the face for 3 to 4 months. The swelling was initially tender but later became nontender and progressively increased in size, approximately 5 to 6 cm in diameter. She gave no history of dysphagia, dyspnea, hoarseness, or significant weight loss. However, she reported occasional blood-tinged sputum over the last 4 to 6 months.

**Figure 1:**
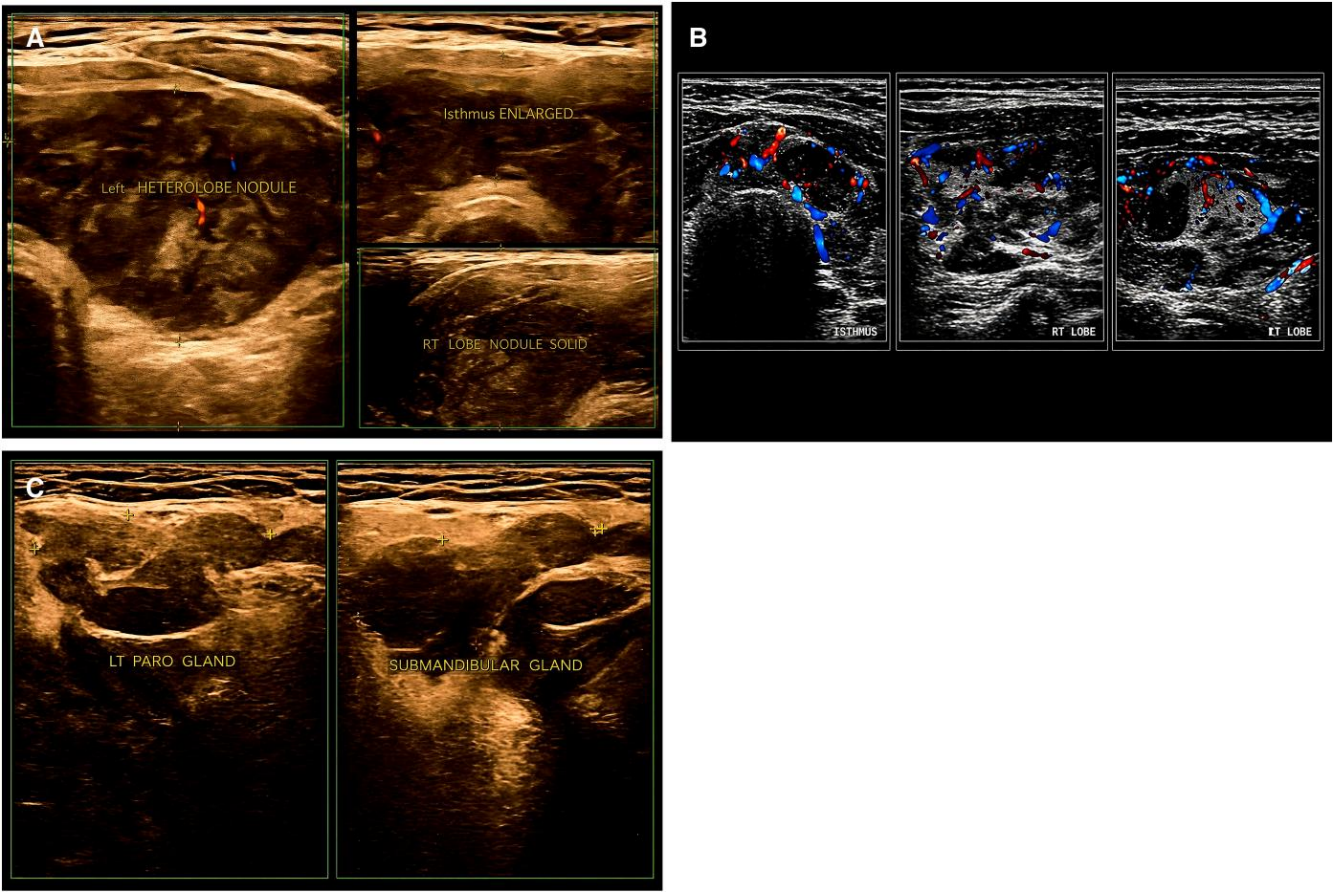
(A) USG of the thyroid gland showing diffuse enlargement and nodules. (B) Color Doppler of the thyroid gland showing increased vascularity. (C) USG showing submandibular gland and parotid gland enlargement.

Her past medical history includes the same type of swelling 10 years previously, and she was diagnosed with hypothyroidism and managed with levothyroxine 75 mcg daily. The swelling then resolved. Three years previously, she again developed swelling and went to a consultant and underwent ultrasonogram (USG) of the neck and fine needle aspiration cytology of the submandibular gland. The USG showed heterogeneous thyroid parenchyma with solid nodules (Fig. 1A) with increased vascularity (Fig. 1B), which is suggestive of Hashimoto thyroiditis, but she did not continue any further treatment as the swelling resolved. She has been hypertensive for about 10 to 15 years, controlled with olmesartan/amlodipine (80 mg/5 mg). She had an episode of mumps and a recent lung infection treated medically. There was no family history of malignancy or thyroid disease, and she did not smoke or drink alcohol.

## Diagnostic assessment

On examination, she was conscious and cooperative with normal vital signs. Neck examination revealed a firm, multinodular, symmetrical thyroid enlargement moving with deglutition. The left submandibular region showed a lobulated, firm swelling along with the tip of the parotid, but nontender. No cervical lymphadenopathy was detected.

Systemic examination was unremarkable except for an occasional cough during deep inspiration. Respiratory auscultation revealed no significant abnormalities.

Investigations showed normal thyroid function tests under medication, but anti-thyroid antibody was 12 530 IU/mL (SI: 12 530 kIU/L) (reference range: <35 IU/mL [SI: <35 kIU]). Also, her random blood glucose level was 335 mg/dL (SI: 18.6 mmol/L) (reference range: 70-140 mg/dL [SI: 3.9-7.8 mmol/L]), glycated hemoglobin (HbA1C) 9.4% (reference range normal: below 5.4%, diabetic: more than 6.5%), and impaired liver function test (alanine aminotransferase [ALT] 179 U/L [SI: 179 U/L]) (reference range: 7-56 U/L [SI: 7-56 U/L]). All the routine investigations were in the reference range. Ultrasonogram of the neck revealed a multinodular goiter with hypoechoic areas suggestive of lymphomatous involvement and submandibular lymphadenopathy and gland enlargement (Fig. 1C). Cytopathology reported a follicular thyroid lesion and chronic sialadenitis.

**Figure 2:**
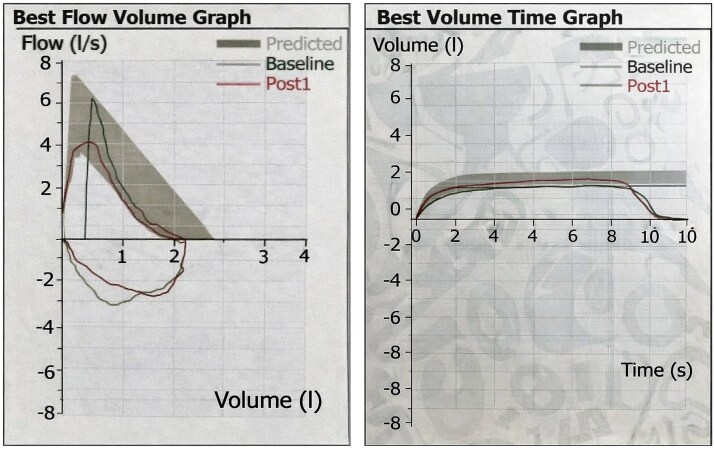
Lung function test showing a restrictive pattern.

Chest X-ray and high-resolution computed tomography of the chest showed bronchiectatic changes, and spirometry indicated a restrictive pattern in Fig. 2. The Mantoux test was negative.

## Treatment

After controlling her uncontrolled diabetes with a combination of short- and long-acting insulin and cough with nebulization and antitussive, a total thyroidectomy was performed under general anesthesia. The postoperative recovery was uneventful, and no thyroid surgery-related complications were observed.

## Outcome and follow-up

**Figure 3:**
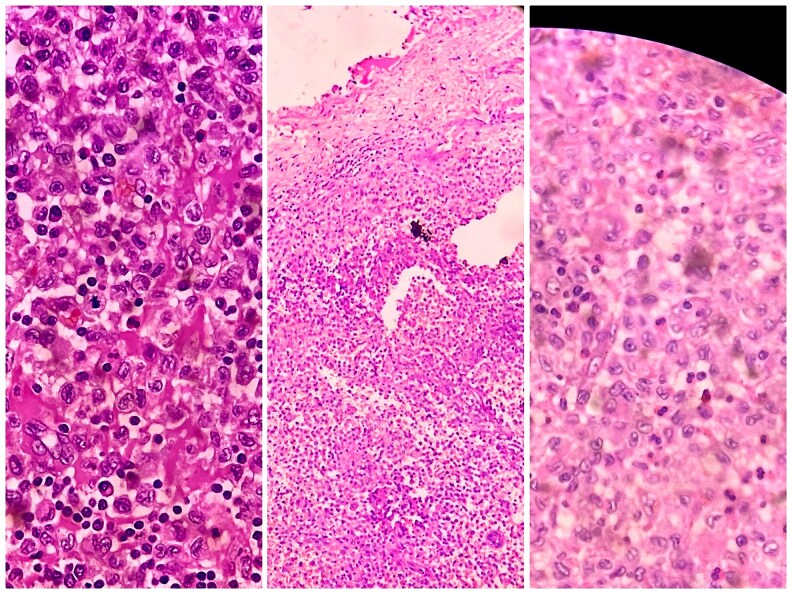
Histopathology of the resected thyroid gland showing infiltration of eosinophils and histiocytoid atypical cells with oval nuclei, prominent nucleoli, and abundant cytoplasm (×100 magnification).

Histopathology of the thyroid gland revealed diffuse infiltration of large atypical lymphoid cells, which may be consistent with LCH or non-Hodgkin lymphoma (likely diffuse large B-cell type), as shown in Fig. 3.

The left submandibular lymph node also showed reactive hyperplasia. However, the immunohistochemistry report showed positive langerin, CD-1a, and CD-68, which was confirmatory for LCH. The postoperative period was uneventful, and the patient was referred to the hemato-oncology department for further management.

## Discussion

The incidence of LCH in adults is approximately 1 to 2 cases per million per year [6]. Thyroid involvement in LCH is rare and can closely mimic other thyroid malignancies, particularly anaplastic carcinoma and primary thyroid lymphoma. The disease encompasses a broad clinical spectrum, ranging from localized (single-system) to disseminated (multisystem) involvement. In multisystem disease, AVP-D is a well-recognized manifestation resulting from pituitary involvement [7, 8].

In the present case, there was no clinical evidence of pituitary or central nervous system involvement, and no features suggestive of AVP-D were observed. However, the patient's newly diagnosed diabetes mellitus raises the possibility of pancreatic involvement, which warrants further evaluation. Thyroid involvement is uncommon in LCH, and concurrent salivary gland involvement, as observed in our case, is exceptionally rare. Clinical presentation may remain asymptomatic for prolonged periods or vary depending on the organs involved. Chronic pulmonary involvement can lead to progressive destruction of lung parenchyma and may eventually manifest as restrictive lung disease, as seen in this case.

Langerhans cell histiocytosis involving the thyroid has been reported in association with other thyroid malignancies, particularly papillary thyroid carcinoma; however, no evidence of coexisting malignancy was identified in our histopathological examination [8]. Although LCH is more commonly encountered in children, it remains rare in adults, contributing to a higher risk of misdiagnosis [7, 8]. Therefore, definitive diagnosis relies on immunohistochemical analysis, typically demonstrating positivity for CD1a, S100, and langerin (CD207). In our case, strong positivity for CD1a and CD68 supported the diagnosis.

The differential diagnoses include Rosai–Dorfman disease, granulomatous thyroiditis, and primary thyroid lymphoma. Accurate recognition of this entity is crucial, as its management and prognosis differ significantly from those of malignant thyroid conditions. Treatment is individualized based on the extent of disease involvement and may range from observation in limited cases to systemic therapy, including corticosteroids, chemotherapy, or targeted therapy such as BRAF inhibitors in *BRAF* V600E-positive cases. Surgical intervention may also be considered in selected cases with thyroid involvement [8, 9]. Prognosis is generally favorable in patients with isolated head and neck disease without systemic involvement.

## Learning points

- This case underscores the diagnostic challenge posed by thyroid LCH mimicking lymphoma (hypothyroidism).
- Immunohistochemistry is essential for accurate diagnosis.
- Awareness of this rare presentation can prevent overtreatment and guide appropriate management.
- Multidisciplinary evaluation remains key in optimizing outcomes for patients with rare head and neck manifestations of LCH.

## Data Availability

Some or all datasets generated during and/or analyzed during the current study are not publicly available but are available from the corresponding author on reasonable request.
